# Circulating (CD3^−^CD19^+^CD20^−^IgD^−^CD27^high^CD38^high^) Plasmablasts: A Promising Cellular Biomarker for Immune Activity for Anti-PLA2R1 Related Membranous Nephropathy?

**DOI:** 10.1155/2016/7651024

**Published:** 2016-07-14

**Authors:** Agnieszka Pozdzik, Ingrid Beukinga, Chunyan Gu-Trantien, Karen Willard-Gallo, Joëlle Nortier, Olivier Pradier

**Affiliations:** ^1^Department of Nephrology, Dialysis and Renal Transplantation, Cliniques Universitaires de Bruxelles (CUB), Erasme Hospital, 1070 Brussels, Belgium; ^2^Unit of Experimental Nephrology, Department of Biochemistry, Faculty of Medicine, Université Libre de Bruxelles (ULB), 1070 Brussels, Belgium; ^3^Department of Hematology, Cliniques Universitaires de Bruxelles (CUB), Erasme Hospital, 1070 Brussels, Belgium; ^4^Unit of Molecular Immunology, Institute Jules Bordet, Université Libre de Bruxelles (ULB), 1000 Brussels, Belgium

## Abstract

Membranous nephropathy (MN) is a kidney specific autoimmune disease mainly mediated by anti-phospholipase A2 receptor 1 autoantibody (PLA2R1 Ab). The adequate assessment of chimeric anti-CD20 monoclonal antibody, rituximab (RTX), efficacy is still needed to improve clinical outcome of patient with MN. We evaluated the modification of plasmablasts (CD3^−^CD19^+^CD20^−^IgD^−^CD27^high^CD38^high^), a useful biomarker of RTX response in other autoimmune diseases, and memory (CD3^−^CD19^+^CD20^+^IgD^−^CD27^+^CD38^−^) and naive (CD3^−^CD19^+^CD20^+^IgD^+^CD27^−^CD38^low^) B cells by fluorescence-activated cell sorter analysis in PLA2R1 related MN in one patient during the 4 years of follow-up after RTX. RTX induced complete disappearance of CD19^+^ B cells, plasmablasts, and memory B cells as soon as day 15. Despite severe CD19^+^ lymphopenia, plasmablasts and memory B cells reemerged early before naive B cells (days 45, 90, and 120, resp.). During the follow-up, plasmablasts decreased more rapidly than memory B cells but still remained elevated as compared to day 0 of RTX. Concomitantly, anti-PLA2R1 Ab increased progressively. Our single case report suggests that, besides monitoring of serum anti-PLA2R1 Ab level, enumeration of circulating plasmablasts and memory B cells represents an attractive and complementary tool to assess immunological activity and efficacy of RTX induced B cells depletion in anti-PLA2R1 Ab related MN.

## 1. Introduction 

Primary membranous nephropathy (MN) is the most common cause of nephrotic syndrome (NS) in Caucasian adults [1]. Recent discovery of autoantibodies (Ab) which recognize specific antigen expressed by podocytes, mainly a soluble receptor of phospholipase A2 (PLA2R1), has greatly improved our understanding of MN physiopathology [2–4]. The anti-PLA2R1 antibody, predominantly the IgG4 subclass, has been reported in sera of nearly 80% of adult MN patients [2, 5, 6]. Accurate cellular immune mechanism(s) involving controlling the synthesis of anti-PLA2R1 Ab still remain(s) unknown [7].

However, the discovery of anti-PLA2R1 Ab highlighted the underestimated role of humoral immunity in MN [9, 10]. Indeed, activated B cells may contribute to the disease progression, not only as effector cells, a precursor of short- and long-lived plasma cells (main cells secreting autoantibodies), but also as regulatory cells of immune response capable of activating T cells [8, 11]. A fraction of plasmablasts (CD3^−^CD19^+^CD20^−^IgD^−^CD27^high^CD38^high^), the intermediate cells between activated B cells and short-lived plasma cells, migrate from secondary lymphoid organs to the bone marrow where they become long-lived plasma cells within the survival niches, a special microenvironment [12]. Plasmablasts produce important cytokines, synthetize antibodies, and act as antigen-presenting cells in inflammatory microenvironment, exhibiting so far underestimated roles in immune regulation [13]. Recently, circulating plasmablasts have been recognized as an early biomarker of immunological activity in autoimmune diseases [14–16].

Reassessment of the pathophysiological involvement of B cells encouraged clinical interest for chimeric anti-CD20 monoclonal antibody (rituximab; RTX) as a more selective treatment modality for PLA2R1 related MN [17–19]. Indeed, RTX is less toxic than actual recommended standard protocols based on corticosteroids and nonspecific immunosuppressants with heavy long-term side effects [20–22]. Series of observational short-term studies have reported the safety and efficacy of RTX alone or in association with other immunosuppressive drugs or plasma exchange in primary as well as in high-risk patients with MN refractory to conventional treatment [17, 23–27]. Discrepancy in dose and treatment duration of RTX, concomitant use of other immunosuppressive drugs, and time of retreatment [9, 10, 28, 29] remain and, unfortunately, relapses and resistance to RTX of anti-PLA2R1 related MN still occur. Indeed, improvement of the clinical outcome of MN is required. Therefore, monitoring of circulating plasmablasts represents an attractive approach to evaluate autoimmune activity and to optimize immunosuppressive therapy in this disease.

To our knowledge, there are no data reporting the time-course of circulating plasmablasts following RTX administration and their relation with circulating anti-PLA2R1 Ab in MN. In this context, we studied the circulating B cells subpopulations by fluorescence-activated cell sorter analysis (FACS) in a single PLA2R1 related MN patient. We looked principally for circulating plasmablasts, memory and naïve B cells, IgG4^+^ B cells, and T regulatory (Treg) cells and we related them to the serum anti-PLA2R1 Ab as well as to proteinuria and glomerular filtration rate (GFR), the current robust kidney clinical endpoints.

## 2. Materials and Methods

**Figure 1:**
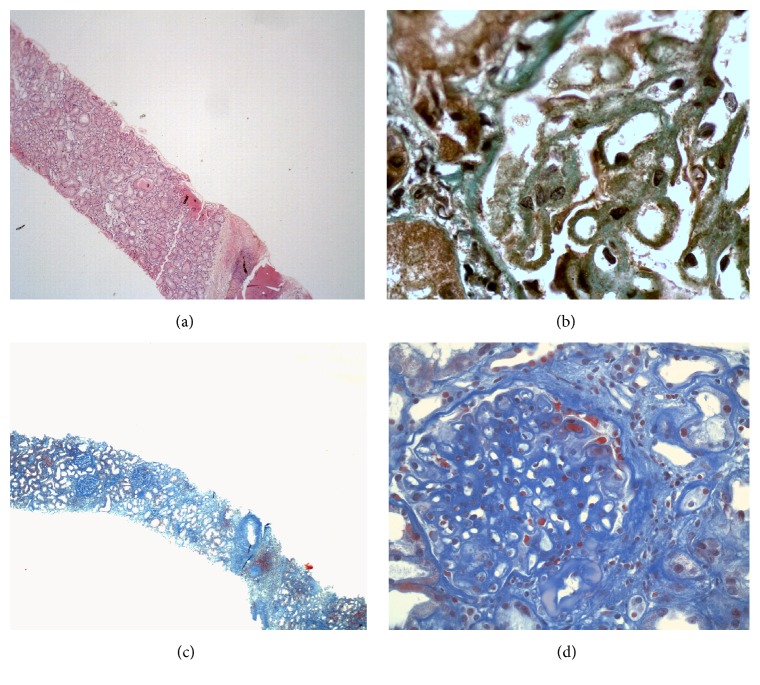
Representative pictures of histopathological analysis performed on formalin-fixed and paraffin embedded kidney tissue section obtained from a first (a-b) and a second kidney punction-biopsy (c-d) performed in patient with membranous nephropathy. (a) Haematoxylin eosin, Jone's silver (b), and Masson Trichrome (c-d) standard stainings. (a) Absence of tubulointerstitial involvement and (b) marked thickening of glomerular basement membrane with stubby spikes-like projections corresponding to stage 2 of membranous nephropathy. (c) Tubular atrophy (tubular dilatation and flattening of epithelium), sparse interstitial inflammation, and fibrosis. (d) Marked thickening of glomerular basement membrane with double contours. Original magnifications: (a) ×40, (b) ×1000 (kindly provided by Dr. Selda Aydin, Pathology Department, St. Luc Hospital, UCL, Brussels, Belgium), (c) ×40, and (d) ×400 (kindly provided by Dr. Michel Depierreux, Pathology Department, Erasme Hospital, CUB, ULB, Brussels, Belgium).

A 48-year-old man presented with nephrotic syndrome and a normal renal function in 1999. Optical and electron microscopy analyses of kidney tissue biopsy were performed in another hospital at that time and showed glomerular lesions typical for membranous nephropathy (Figures 1(a) and 1(b)). Screenings for secondary immunological causes including antinuclear antibody, rheumatoid factor, anti-neutrophil cytoplasmic antibody, and serology for virus hepatitis B and C were negative. Complement level was normal. Secondary drug-induced MN was evoked considering chronic use of nonsteroidal anti-inflammatory drug. Nephroprotection was started.

### 2.1. Assessment of Peripheral T and B Cells Subpopulations, IgG4^+^ B Cells, and Plasmablasts

In our case, we performed a peripheral blood analysis during a 5-year follow-up under three different immunosuppressive therapies: firstly under MPD in association with TRL, secondly under MMF alone, and finally under RTX. We started to study only CD3^+^CD4^+^/CD3^+^CD8^+^ T cells ratio and CD19^+^ B cells under MPD + TRL and MMF by standardized routine protocol. Concomitantly, we develloped the analysis of B cells subpopulations. Briefly, whole blood samples (5 mL EDTA anticoagulated) were received at the hematology laboratory and WBC count was performed on UniCel DxH*™* 800 hematology analyzers from Beckman Coulter within 4 hours. In the flow cytometry laboratory, 2 mL blood was washed three times with PBS-1% BSA to remove plasma. The white and red cells pellet was suspended vol/vol in PBS-1% BSA and 100 *μ*L was stained with 20 *μ*L monoclonal antibody combinations: IgD-FITC (DakoCytomation, Heverlee, Belgium), CD27-PE, CD10-ECD, CD5-PE-Cy5.5, CD19 PE-Cy7, CD23-APC-AF700, CD38-APC-AF750, CD20 Pacific blue, and CD45 Krome Orange (all from Beckman Coulter). T, NK, and monocyte markers were added in the same ratio to negatively purify the B cell populations. Staining was performed during 15 min in the dark and the cell suspension was washed once with PBS-1% BSA. The B and plasma cells fluorescence was acquired with a 10-color Beckman Coulter Navios flow cytometer driven by CXP-Navios software. At least 4000 CD19 positive cells were acquired. Analysis was performed using Kaluza 1.2 Software (Beckman Coulter). Mature B cells were defined as CD19^−^CD20^−^CD45^bright^ positive cells, naive B cells as IgD^+^, and CD38^dim^ (resting naive), CD38^+^ (activated naive), and memory cells as IgD^−^ with CD38^dim^ or CD38^+^. Transitional B cells were isolated as CD38^bright^, CD20^+^, CD19^+^ IgD^+^, and CD27^−^ cells and finally plasmablasts were CD45^+^ but not bright, CD19^+^, CD20^−^, CD38^bright^, CD27^+^, and IgD^−^. Circulating B cells' absolute values were calculated in double platform using absolute lymphocyte count from the hematological analyzer associated with the B cell population percentages.

### 2.2. Detection of Circulating Anti-PLA2R1 Antibodies

Circulating antibodies against PLA2R1 were assessed by commercially available cell based assay using indirect immunofluorescence using human embryonic kidney (HEK-293) cells transfected with human PLA2R1 (Euroimmun, Lubeck, Netherlands) [30] and by commercially available PLA2R1 ELISA kit (Biognost, Heule, Belgium) according to the manufacturer's instructions as reported by others [6, 30, 31].

### 2.3. Analysis of Renal Biopsy Specimens

The specimens from both kidney biopsies were prepared for light and immunofluorescence microscopy by standard technique as previously described [32].

### 2.4. Detection of PLA2R1 Antigen in Paraffin Embedded Kidney Biopsy

Both kidney biopsies were assessed for localization of PLA2R1 in glomerular deposits. We used rabbit affinity purified specific anti-PLA2R1 antibodies (Atlas Antibodies, AB, Stockholm, Sweden) followed by goat FITC-conjugated anti rabbit Fab IgG as previously reported [33].

### 2.5. Detection of Macrophages, T and B Cells

Only the second kidney biopsy was available for immunophenotyping of intrarenal macrophages, T and B cells. We performed immunohistochemistry using the following anti-human primary antibodies: CD68 (macrophages), CD8 (CD8^+^ T cells subpopulation), CD4 (CD4^+^ T cells subpopulation), and CD20 (mature B cells) as described previously [32].

### 2.6. Detection of Intrarenal Follicular Dendritic Cells

Only second kidney biopsy was available for immunofluorescence staining that was adapted from Gu-Trantien et al. [34]. We used the following primary antibodies: CD20 (ab9475, 1/50), CXCL13 (AF801, 1/200), and CD21 (ab75985, 1/500) and donkey anti-mouse (ab98767, 1/200), anti-goat (ab98514, 1/200), and anti-rabbit (ab98491, 1/200) secondary antibodies. Except for CXCL13 obtained from R&D System (Abingdon, UK), all were provided from Abcam (Cambridge, UK). Briefly, after paraffin removal with xylene, mounted sections were pretreated (citrate 10 mM, pH 6.0, at 95°C to 99°C for 30 minutes), blocked with 1% BSA for 30 min, and incubated with primary antibodies (at 4°C, moist chamber, overnight). After washing, secondary antibodies were applied for 2 h at room temperature. Slides were mounted in the ProLong Gold Antifade Mountant with DAPI (P36941, Life Technologies, California, USA) and images were acquired with a Zeiss LSM 710 confocal microscope.

## 3. Results 

**Figure 2:**
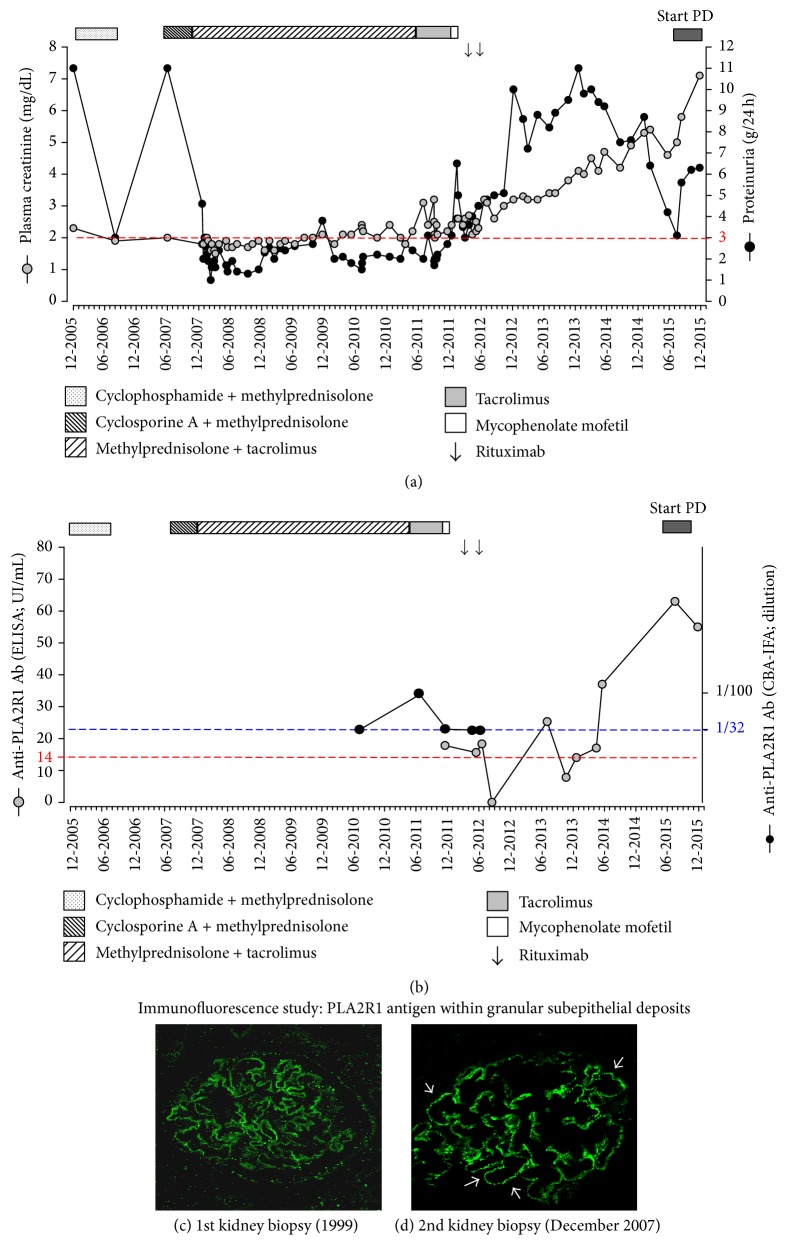
Time-course of renal function parameters (a) plasma creatinine and 24 h-proteinuria levels under immunosuppressive therapy and (b) values of serum anti-PLA2R1 antibody levels. (c and d) Representative pictures of immunofluorescence of phospholipase A2 receptor expression within the glomerulus performed on formalin-fixed and paraffin embedded kidney tissue section obtained from a first (c) and a second (d) kidney biopsy in patient with membranous nephropathy. Confocal microscopic analysis of a paraffin kidney biopsy specimen revealed the presence of PLA2R1 in subepithelial deposits along glomerular capillary loops (white arrows), ×400 magnification. Kindly performed by Hanna Debiec INSERM U702, Hôpital Tenon, Paris, France.

In December 2005, because of uncontrolled nephrotic proteinuria [urinary proteins (UPr) 11.0 g/24 h] and renal function decline, cyclophosphamide and methylprednisolone (MPD) were given according to Ponticelli revised protocol. Only partial remission was obtained (UPr 3.0 g/24 h) (Figure 2(a)). In June 2007, considering relapse of NS (UPr 11.0 g/24 h), cyclosporine A and MPD (275 mg and 12 mg daily, resp.) were started but, due to drop in proteinuria, remained below the nephrotic range. At the time of admission in our nephrology clinic in November 2007, we noticed uncontrolled NS (lower limbs edema, uncontrolled arterial hypertension, and dyslipidemia). In December 2007, proteinuria remained nephrotic (UPr 4.0 g/24 h). In order to exclude the lesions of focal segmental glomerulosclerosis (FSGS) secondary to chronic proteinuria, kidney biopsy was performed and showed only advanced stage of MN without FSGS, mild tubular atrophy, and renal interstitial fibrosis associated with mononuclear cells interstitial infiltration (Figures 1(c) and 1(d)). Then, we switched immunosuppression to the association of tacrolimus (TRL) with MPD because of cyclosporine A adverse effects, mainly important gingival hypertrophy. In July 2011, considering acute kidney injury (stage 3) secondary to a viral gastroenteritis (Figure 2(a)), we removed sartans because of uncontrolled hyperkaliemia and reduced MPD (2 mg daily) because of long-term corticoids adverse effects (partial rupture of left biceps). As renal function declined progressively, we started mycophenolate mofetil (1500 mg daily) in March 2012.

During early pretransplant, we retrospectively screened both kidney biopsies taken in 1999 and in 2007 by immunofluorescence (Figures 2(c) and 2(d)). The presence of several granular PLA2R1 antigens within extramembranous deposits was demonstrated in only one but well-preserved glomerulus in tissue provided from a first kidney biopsy (1999). Kidney biopsies contained ineffaceable fingerprints of anti-PLA2R1 autoimmunity also in several glomeruli provided from second biopsy (after 7 years of well-conducted immunosuppression) and confirmed primary MN.

Unfortunately, the serum was unavailable at the moment of MN diagnosis. Available serum samples from 2007 to 2010 were negative for anti-PLA2R1 Ab (sensitive technique, cell based assay using indirect immunofluorescence (CBA-IFA), a reference technique for diagnosis of MN [6, 31, 35, 36]). In 2012, after removal of immunosuppression, we detected low levels of circulating anti-PLA2R1 Ab as measured by CBA-IFA and by enzyme-linked immunoabsorbent assay (Figure 2(b)). Our long-term data reinforce the importance of methodological screening of renal tissues and repeated serum assessment for PLA2R1 staining within extramembranous deposits and for circulating anti-PLA2R1 Ab, respectively, in the management of IMN.

In May 2012, we gave RTX (two doses of 375 mg/m^2^ every 2 weeks, off label use, low doses as proposed previously) according to promising data form Cravedi et al. [37] and MPD (4 mg daily, to prevent anti CD20-antibody immunization) in PLA2R1 related MN in an attempt to control NS and CKD progression. Despite targeted CD19^+^ cells, lymphopenia and proteinuria remained in the nephrotic range and the renal function deteriorated progressively (PrU 6.0 g/24 h, PCr 3.5 mg/dL). We withdrew immunosuppression and addressed the patient to the predialysis unit. For the moment, he is doing well on peritoneal dialysis and he is on the waiting list for kidney transplantation. However, as the level of circulating anti-PLA2R1 Ab remains high (66 UI/mL, normal range 0–20 UI/mL), RTX will be administrated in order to reduce the levels of Ab.

The number of CD19^+^ B cells was not affected by MPD + TRL but was lower than normal range under MMF. After one dose of RTX, CD19^+^ B cells decreased rapidly (<5 cells/*μ*L) and were fully cleared at day 30 (data not shown).

**Figure 3:**
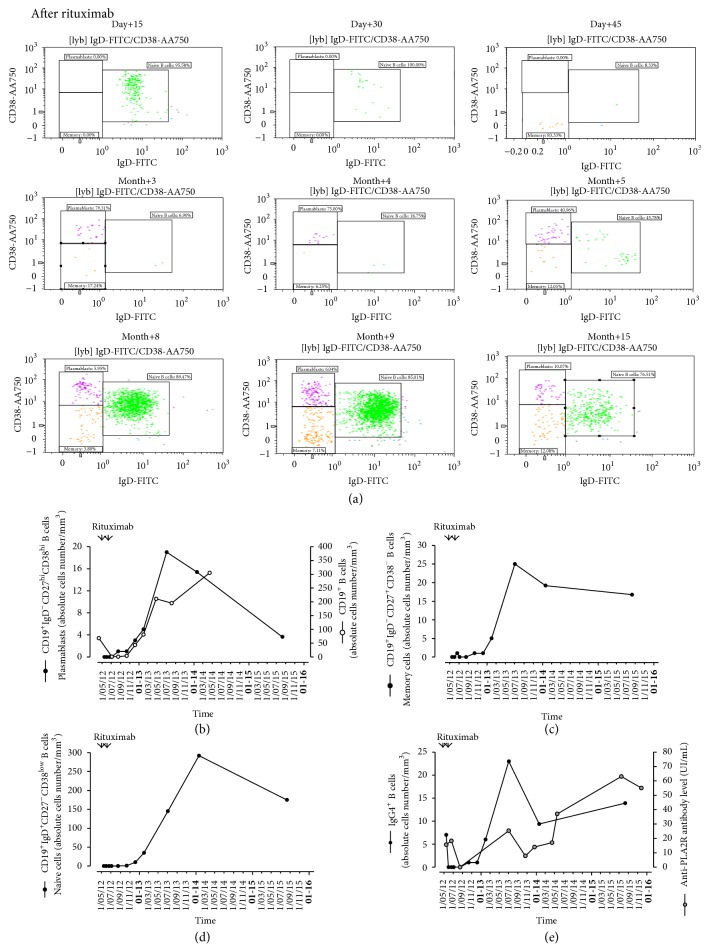
Distribution of the B lymphocyte subpopulations after B cells depleting therapy with rituximab as evaluated by fluorescence-activated cell sorter (FACS) analysis of peripheral blood mononuclear cells (PBMC). (a) Dot-plots representing the expression of IgD (FITC) and CD38 (AA750) monitored in our patient with corticosteroid-resistant PLA2R1 related membranous nephropathy during 15 months after first rituximab injection. Graphs representing the kinetics of absolute cells number per mm^3^ of (b) plasmablasts (CD3^−^CD19^+^CD20^−^IgD^−^CD27^high^CD38^high^), (c) memory B cells (CD3^−^CD19^+^CD20^+^IgD^−^CD27^+^CD38^−^), and (d) naïve (CD3^−^CD19^+^CD20^+^IgD^+^CD27^−^CD38^low^) B cells. (e) IgG4^+^ B cells (absolute cells number per mm^3^) and anti-PLA2R1 antibody assessed in serum expressed in IU/mL.

All subpopulations of circulating B cells: (CD3^−^CD19^+^CD20^+^IgD^−^CD27^+^CD38^−^) memory B cells, plasmablasts (CD3^−^CD19^+^CD20^−^IgD^−^CD27^high^CD38^high^), and naïve B cells (CD3^−^CD19^+^CD20^+^IgD^+^CD27^−^CD38^low^) (Figure 3(a)) were affected. Plasmablasts, memory B cells were cleared at day 15 and naive B cells at day 45. Despite persisting CD19^+^ lymphopenia, as soon as 45 days after first RTX injection, memory B cells, plasmablasts, and naive B cells repopulated peripheral blood (Figures 3(b)–3(d)). After a transient pick (14 months), the number of circulating plasmablasts decreased progressively. However, the number of memory B cells remained elevated.

Because anti-PLA2R1 antibody is related mainly to IgG4 subclass, we investigated circulating IgG4^+^ B cells. These B cells disappeared after RTX and their reappearance followed kinetics of circulating plasmablasts. Moreover, circulating anti-PLA2R1 antibodies followed the increase in circulating plasmablasts, memory B cells, and IgG4^+^ B cells (Figures 3(b)–3(e)).

Peripheral CD4^+^CD25^high^CD127^dim^FoxP3^+^ regulatory T cells (Treg) were also affected by RTX (data not shown). The peripheral Treg cells progressively increased after RTX administration.

**Figure 4:**
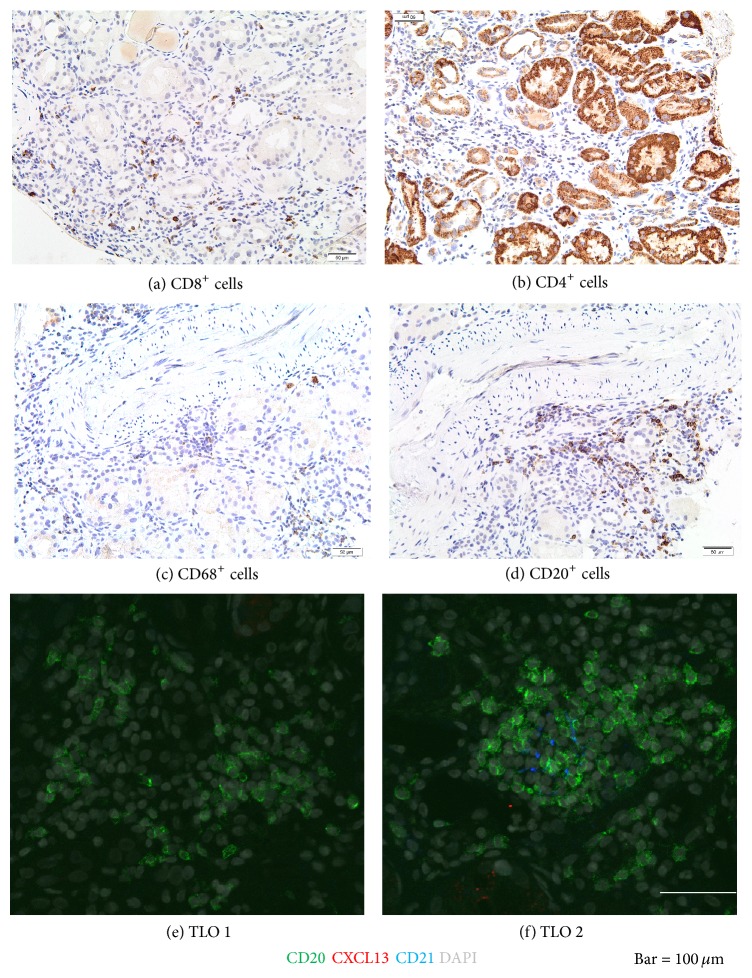
Representative pictures of intrarenal inflammatory cells identified by immunoperoxidase and double immunofluorescence stainings in tissue samples provided from second kidney biopsy. Kidney tissue sections were probed with antibodies against the following antigens: CD8 (cytotoxic T cells), CD4 (helper/suppressor T cells), CD68 (monocytes/macrophages), and CD20 (B cells). One of the two small B cell follicles, which corresponded to tertiary lymphoid organs contained CD21+ follicular dendritic cells indicating the presence of germinal center activities despite the absence of the B cell attracting chemokine, CXCL13 within the germinal center. Original magnifications: (a–d) ×200 and (e-f) ×1000.

Furthermore, we performed immunohistochemical stainings of renal tissue from the second kidney biopsy to characterize intrarenal inflammatory cells. Several CD8^+^, CD4^+^, and CD68^+^ cells infiltrated interstitial areas diffusely, reflecting renal interstitium infiltration by T and B cells as well as the presence of monocytes/macrophages, respectively (Figures 4(a)–4(d)). Several CD20^+^ cells formed rather patchy, focal infiltrate in peritubular areas. Interestingly, one of the three small B cell intrarenal follicles detected on the biopsy sample contains some CD21^+^ follicular dendritic cells (FDCs) (Figures 4(e) and 4(f)) indicating germinal center (GC) corresponding to tertiary lymphoid organ (TLO), despite absence of the B cells attracting chemokine CXCL13 within the GC.

## 4. Discussion

To our knowledge, this is the first report of long-term modification in circulating B cell subtypes including plasmablasts (CD3^−^CD19^+^CD20^−^IgD^−^CD27^high^CD38^high^) and memory (CD3^−^CD19^+^CD20^+^IgD^−^CD27^+^CD38^−^) and naive (CD3^−^CD19^+^CD20^+^IgD^+^CD27^−^CD38^low^) B cells in relation to serum level of anti-PLA2R1 Ab, proteinuria, and GFR.

We suggest that circulating CD3^−^CD19^+^CD20^−^IgD^−^CD27^high^CD38^high^ plasmablasts could be a new cellular biomarker of residual autoimmunity in PLA2R1 related MN. We argue our suggestion considering (1) the serum anti-PLA2R1 antibody, a proposed biomarker of humoral activity in this disease, (2) the evidence of marked CD20^+^ B cells infiltration, and (3) the formation of tertiary lymphoid organ by CD21^+^ follicular dendritic cells (FDCs) indicating germinal center (GC) activities in our case.

However, we fully recognize the following limitations of our study: firstly, this being a one-case study; secondly, the late stage of disease; and thirdly, the possibility that baseline number of plasmablasts before RTX could have been influenced by previous immunosuppression.

We hypothesized that previous immunosuppressive drugs blocked anti-PLA2R1 Ab synthesis but were unable to clear it away from glomerular deposits and/or to remove intrarenal TLO hypothetical site of B cells activation. In our case, after short disappearance induced by RTX, serum PLA2R1 Ab became positive, probably secondary to immunosuppression withdrawal, and correlated with progressive increase in PCr and proteinuria. Failure of RTX in our patient could be explained at least by the following reasons: (1) low levels of anti-PLA2R1 Ab (slightly higher than the normal range) as RTX has been reported to preserve residual renal function in patients, especially in those with high levels; (2) development of glomerulosclerosis, which is always possible with such a long history of active MN and which was recognized previously to be associated with poorer outcome under RTX therapy [38] or/and secondary tubulointerstitial lesion considering the many years of calcineurin inhibitors use; (3) the possibility that AKI episode could also be involved as cytokines and/or chemokines from injured tubular renal cells, which are able to induce autoimmune B cells response [39]. Indeed, we found marked intrarenal infiltration by CD8^+^ and CD4^+^ cells, which has been associated with pejorative evolution of MN [40]. In both human and experimental models of MN, the Th2-polarization of CD4^+^ T cells stimulated intrarenal autoreactive CD20^+^ B cells to generate IgG4 [41]. We observed several CD20^+^ B cells that exclusively infiltrated the tubulointerstitium, but not the glomerular tuft. However, no relation has been found between the response to RTX and CD20^+^ cells in the kidney biopsy in MN [42].

In our case, RTX induced a complete depletion of all peripheral B cells subpopulations (naive, transitional, memory B cells, and plasmablasts) but circulating plasmablasts appeared first despite of achieved CD19^+^ depletion and were followed by an increase in serum anti-PLA2R1. Recent evidence suggests that plasmablasts produce cytokines, synthetize antibodies, and act as antigen-presenting cells especially in chronically inflamed organ [12]. Increase in percentage of IgD^−^CD27^high^ plasmablasts has been associated with high anti-ds DNA antibody levels relapse in SLE patient [43]. Circulating plasmablast counts have been proposed as a useful biomarker of residual autoimmunity assessing response to treatment and determining the appropriate time to retreatment independently of IgG4 serum concentration in the IgG4-related disease [44]. Tissue plasmablasts have been recognized as a pivotal predictor of clinical outcome in patients receiving RTX for refractory rheumatoid arthritis [45]. However, RTX induced B cell deletion promotes a suitable microenvironment for maturation and survival of autoimmune long-lived plasma cells in the spleen [46]. Accordingly to Cohen et al. [47], higher doses or longer duration of RTX could be necessary to control circulating plasmablasts in MN. Despite the fact that origin and localization of B cells producing anti-PLA2R1 Ab remain unknown, previously proposed titration of RTX to circulating CD20^+^ or CD19^+^ B cells needs to be revised in MN as not measuring diversity of B cells subpopulations [48]. Indeed, level of plasmablasts, rather than the criterion of complete depletion of peripheral CD20^+^ or CD19^+^ B cells after RTX administration, predicted the RTX success in rheumatoid arthritis [49]. It could be speculated that circulating plasmablasts originate from activated intrarenal B cells within remaining TLO considering that tissue B cells are difficult to deplete by RTX [16], but this hypothesis needs to be proven. Clearly, further clinical long-term follow-up studies of MN patients under immunosuppression are indispensable to investigate the usefulness of B cells subsets monitoring to improve the assessment of autoimmunity in MN. We need the responses to 2 questions: (1) Are the circulating plasmablasts a more specific marker of residual autoimmunity in MN? (2) Is resistance to immunosuppression related to the persistence of long-lived plasma cells within intrarenal TLO?

In conclusion, measurement of circulating (CD3^−^CD19^+^CD20^−^IgD^−^CD27^high^CD38^high^) plasmablasts rapidly assesses RTX response and appears as a promising cellular biomarker to improve RTX therapy in clinical practice. We suggest that besides monitoring of serum anti-PLA2R1 Ab level, enumeration of plasmablasts and memory B cells represents an attractive and complementary tool to assess autoimmune activity and/or efficacy of RTX induced B cells depletion in anti-PLA2R1 Ab related MN.

## Abbreviations

CKD: Chronic Kidney Disease
PCr: Plasma creatinine
anti-PLA2R1 Ab: Anti-phospholipase A2 receptor type 1 antibody
MN: Membranous nephropathy
NS: Nephrotic syndrome
ESKD: End Stage Kidney Disease
TLO: Tertiary lymphoid organ.
